# PDLIM4 promotes dephosphorylation of STAT transcription factors by recruiting PTP-BL and inhibits Th1, Th2, and Th17 cell differentiation

**DOI:** 10.1093/intimm/dxag019

**Published:** 2026-05-25

**Authors:** Aya Jodo, Masakiyo Nakahira, Yuta Kochi, Akiko Sugimoto-Ishige, Tsuneyasu Kaisho, Takashi Tanaka

**Affiliations:** Laboratory for Inflammatory Regulation, RIKEN Center for Integrative Medical Sciences, Yokohama, Kanagawa 230-0045, Japan; Department of Immunology, School of Medicine, Hyogo Medical University, Nishinomiya, Hyogo 663-8501, Japan; Department of Genomic Function and Diversity, Medical Research Laboratory, Institute of Integrated Research, Institute of Science Tokyo, Tokyo 113-8510, Japan; Laboratory for Autoimmune Diseases, RIKEN Center for Integrative Medical Sciences, Yokohama, Kanagawa 230-0045, Japan; Laboratory for Inflammatory Regulation, RIKEN Center for Integrative Medical Sciences, Yokohama, Kanagawa 230-0045, Japan; Industry-Government-Academia Collaboration Promotion Headquarters, Wakayama Medical University, Wakayama, Wakayama 641-8509, Japan; Laboratory for Inflammatory Regulation, RIKEN Center for Integrative Medical Sciences, Yokohama, Kanagawa 230-0045, Japan

**Keywords:** LIM domain, protein tyrosine phosphatase, T-helper cell

## Abstract

STAT (signal transducers and activators of transcription) transcription factors are activated by tyrosine phosphorylation after cytokine stimulation and are critical for the differentiation of T-helper (Th) cells into particular Th lineage subsets. How STAT-mediated Th cell differentiation is negatively regulated, however, is not fully understood. Here, we report that PDLIM4 binds to STAT3, 4, and 6 and suppresses gene activation mediated by these STATs. PDLIM4 acts as an adaptor that recruits PTP-BL, a protein tyrosine phosphatase, through its LIM (abnormal cell lineage 11-islet 1-mechanosensory abnormal 3) domain, facilitating dephosphorylation of STAT proteins. PDLIM4-deficiency in CD4^+^ T cells resulted in augmented tyrosine phosphorylation of these STAT proteins and consequently enhanced Th1, Th2, and Th17 cell differentiation, suggesting that PDLIM4 regulates the differentiation of multiple lineages of Th cells by suppressing STAT signaling. We further found that a non-synonymous single-nucleotide polymorphism in *PDLIM4*, which causes the substitution of a glycine residue with a cysteine in the LIM domain, is associated with susceptibility to rheumatoid arthritis and Graves’ disease, both of which are known to be Th17 cell-driven autoimmune diseases. Notably, PDLIM4 containing this amino acid substitution in the LIM domain showed reduced binding to PTP-BL and was therefore partially impaired in its ability to dephosphorylate STAT3 and suppress STAT3 signaling. Our findings define an essential role of PDLIM4 in negatively regulating STAT-mediated Th-cell differentiation and preventing the onset of human autoimmune diseases.

## Teaser

PDLIM4 negatively regulates Th-cell subset differentiation

## Graphical Abstract

**Graphical Abstract:**
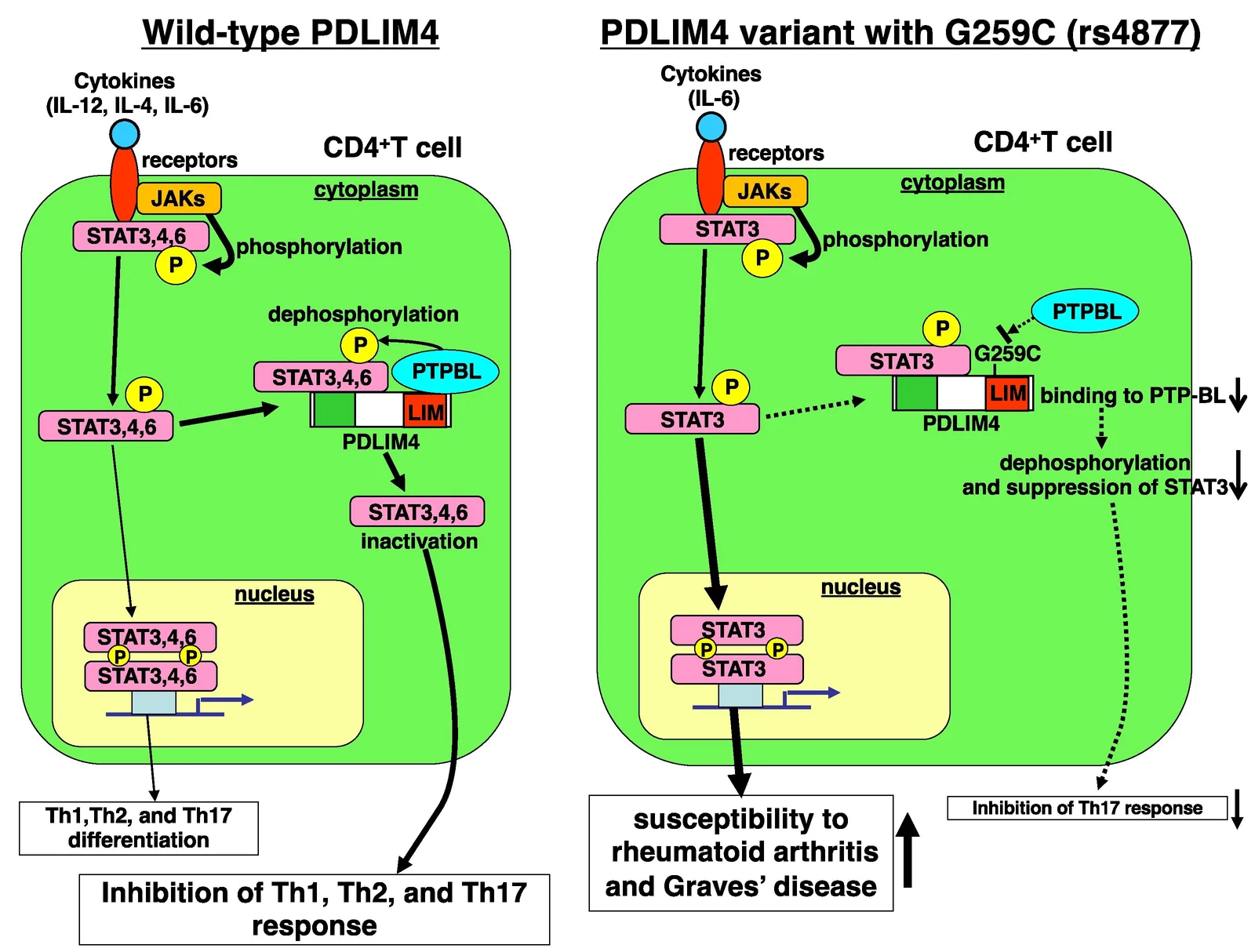

## Introduction

CD4^+^ T cells play central roles in host immune and inflammatory responses (1). Depending on the invading pathogens, stimulation with different cytokines activates naïve CD4^+^ T cells and directs them to differentiate into distinct T cell subsets, T-helper (Th) 1, Th2, and Th17 cells to fight against a variety of microorganisms. Signal transducers and activators of transcription (STAT) transcription factors, STAT4, STAT6, and STAT3, are critical for the differentiation of Th1, Th2, and Th17 cells, respectively. STAT family proteins are latent cytoplasmic transcription factors whose activation is strictly regulated by cytokine stimulation. Upon binding of IL-6, IL-12, or IL-4 to their receptors, STAT3, STAT4, and STAT6 become tyrosine phosphorylated, respectively, by receptor-associated JAK family kinases. Phosphorylated STAT proteins then form dimers, translocate into the nucleus, and induce the expression of Th cell differentiation-related genes (2). The differentiation and activation of Th cells is tightly regulated, otherwise exaggerated Th cell-mediated responses may cause autoimmune and inflammatory diseases, such as rheumatoid arthritis and bronchial asthma (2, 3). How STAT-mediated Th cell differentiation is negatively regulated, however, is not fully understood.

Several factors have been reported to interact with STAT proteins and inhibit their activity. For example, some protein tyrosine phosphatases directly dephosphorylate activated STAT proteins (4). These include TC-PTP for STAT1 (5), STAT3 (6), and STAT6 (7); PTP1B for STAT3 (8) and STAT6 (9); SHP2 for STAT3 (10) and STAT5 (11); PTP-BL for STAT4 and STAT6 (12), and LMW-DSP2 for STAT3 (13). On the other hand, the protein inhibitor of activated STAT (PIAS) family of proteins blocks STAT-DNA binding, thereby suppressing STAT-mediated gene activation (14, 15). In addition, we previously demonstrated that PDLIM2, a nuclear protein that contains both PDZ (postsynaptic density 65-discs large-zonula occludens 1) and LIM (abnormal cell lineage 11-islet 1-mechanosensory abnormal 3) domains, negatively regulated both STAT4-mediated Th1 differentiation and STAT3-mediated Th17 differentiation. As a ubiquitin E3 ligase of these STAT proteins in CD4^+^ T cells, PDLIM2 bound to STAT3 and STAT4 and then promoted their polyubiquitination and proteasome-dependent degradation, thereby suppressing STAT3- and STAT4-mediated signaling (16, 17). Consistently, PDLIM2 deficiency resulted in enhanced Th1 and Th17 cell differentiation. PDLIM2 belongs to a large family of LIM proteins; to date, 58 LIM proteins have been identified and are classified into several subgroups based on differences in their domain structures (18, 19). LIM proteins that have a PDZ domain at the N-terminus and a LIM domain at the C-terminus, like PDLIM2, are defined as the PDZ-LIM protein subfamily, which consists of PDLIM1/Elfin, PDLIM2/SLIM/Mistique, PDLIM3/ALP, PDLIM4/Ril, PDLIM5/ENH, PDLIM6/ZASP/Cypher, and PDLIM7/Enigma/LMP1 (20). Since both PDZ and LIM domains are involved in protein-protein interactions, PDZ-LIM proteins are thought to function as adaptor proteins that regulate the functions of target proteins by binding to and recruiting active intracellular molecules via their LIM and PDZ domains (18, 19). However, their functions in the immune system mostly remained unclear. We predicted that PDZ-LIM proteins could constitute a new family that negatively regulates immune responses and have attempted to clarify the role of these proteins in the immune regulation. We have reported that PDLIM2 and PDLIM7 negatively regulated NF-κB-mediated inflammatory responses in dendritic cells as ubiquitin E3 ligases for the p65 subunit of the NF-κB transcription factor. PDLIM2 and PDLIM7 bound to p65 and promoted its polyubiquitination and proteasome-dependent degradation (21, 22). Moreover, PDLIM1, a cytoplasmic LIM protein, bound to and sequestered p65 in the cytoplasm by interaction with actin stress fibers through binding to α-actinin via its PDZ domain, thereby suppressing its nuclear translocation (23). However, the role of other PDZ-LIM proteins besides PDLIM2 in the regulation of CD4^+^ T cells remains unknown. In this study, we focused on the analysis of PDLIM4, the LIM protein most structurally related to PDLIM2. Since PDZ and LIM domains are protein-binding modules, previous studies of PDLIM4 have been focused on the identification of its binding partners and have shown that it is involved in the regulation of cytoskeleton organization and oncogenesis (24–27). Here we have demonstrated that PDLIM4, which is located in the cytoplasm, binds to STAT3, STAT4, and STAT6 and negatively regulates Th1, Th2, and Th17 cell differentiation. Mechanistically, PDLIM4 associated with and recruited PTP-BL, a protein tyrosine phosphatase, through its LIM domain, and facilitated dephosphorylation of the tyrosine residue of STAT3, STAT4, and STAT6, thereby suppressing STAT3-, STAT4-, and STAT6-mediated signaling. In CD4^+^ T cells, PDLIM4-deficiency resulted in augmented tyrosine phosphorylation of these STAT proteins and consequently enhanced Th1, Th2, and Th17 cell differentiation. We further found that a non-synonymous single-nucleotide polymorphism (nsSNP) in *PDLIM4* is associated with rheumatoid arthritis susceptibility (rs4877; odds ratio 1.13, *P* = 0.0041). This SNP, found at 30% in the Japanese population, causes the substitution of a glycine residue in the LIM domain with a cysteine. Interestingly, the PDLIM4 mutant containing this amino acid substitution in the LIM domain showed reduced binding to PTP-BL, and therefore partially impaired STAT3 dephosphorylation and suppression of STAT3 signaling. These data reveal that PDLIM4 is crucial for negatively regulating STAT-mediated Th-cell differentiation and preventing the onset of human autoimmune diseases.

## Methods

### Expression plasmids

For His- and c-Myc-tagged PDLIM4, mouse *Pdlim4* (GenBank accession: NM_019417) was subcloned into pcDNA3-His (Invitrogen) and pCMV-Myc (Clontech), respectively. The frameshift mutant (FS) was prepared by digestion of the *Pdlim4* cDNA with BstEII, followed by self-ligation to introduce a frameshift immediately after the 5′ end of the reading frame. For PDLIM4 mutants lacking the LIM (ΔLIM) or PDZ domains (ΔPDZ), cDNAs corresponding to residues 1–246 or 100–330 of *Pdlim4* were subcloned into pCMV-Myc. For the G259C PDLIM4 mutant, Gly-259 was replaced with Cys using a QuikChange Lightning Site-Directed Mutagenesis Kit (Agilent Technologies). Flag-tagged murine STAT4 and untagged-human STAT6 were cloned into pcDNA3. Flag-tagged murine STAT3 was in pRc/CMV. C-Myc-tagged PTP-BL and its substrate- trapping mutant (PTP-BL-ST), in which a C2285S mutation was introduced to inactivate phosphatase activity, were kindly provided by M. J. Grusby (Harvard School of Public Health, Boston, MA, USA) (12). For c-Myc-tagged PTP1B, TC-PTP, LMW-DSP2, and SHP2, the coding regions of mouse *Ptpn1* (GenBank accession: NM_011201), *Ptpn2* (GenBank accession: NM_008977), *Dusp22* (GenBank accession: NM_001037955), and *Shp2* (GenBank accession: NM_011202) were subcloned into pCMV-Myc. The c-Myc-tagged PDLIM2 was previously described (16). The (2x) IRF-1 luciferase reporter plasmid, containing two copies of a high-affinity STAT binding site from the promoter region of the IRF-1 gene, and the STAT6-responsive luciferase reporter plasmid, containing STAT6 and C/EBP binding sites from the human immunoglobulin heavy-chain germ line ε promoter, were previously described (12). The pRL-Null renilla construct (#E2271) was purchased from Promega.

### Reagents and antibodies

Murine IL-4, IL-6, IL-12, and human IFN-α and IL-4 were purchased from R&D Systems. Human TGF-β was purchased from Wako Pure Chemical Industries. STAT4 (sc-486), Tyk2 (sc-169), PKC (sc-10800), Sp1 (sc-59), HSP90 (sc-7947) antibodies, and Omni-probe (sc-499; used as an anti-His antibody) were purchased from Santa Cruz Biotechnology. STAT3 (#9132), STAT6 (#5397), phospho-STAT3 (#9145), JAK1 (#3344), phospho-Tyk2 (Tyr1054/1055) (#68790), LSD1 (#2184), Cdc37 (#3604), and β-actin (#4970) antibodies were purchased from Cell Signaling Technology. A DYKDDDDK (NU01102) antibody was purchased from Nacalai USA and used as an anti-Flag antibody. The HRP-conjugated c-Myc antibody (M047-7), c-Myc antibody-conjugated agarose (M047-8), and DDDDK-tagged Protein PURIFICATION GEL (#3328R) (used for immunoprecipitation of Flag-tagged proteins) were purchased from MBL. Anti-phosphotyrosine was purchased from Millipore (#05-1050; 4G10). The ubiquitin antibody (clone FK-2; BML-PW8810) was purchased from Enzo Life Sciences. Anti-PDLIM4 (Anti-Ril; ab1555509) was purchased from Abcam. As secondary antibodies, HRP-goat anti-rabbit IgG (#111-035-003) was purchased from Jackson ImmunoResearch, and HRP-sheep anti-mouse IgG (NA931) was purchased from GE Healthcare. CD3 (145-2C11) and CD28 (37.51) were purchased from BD Biosciences. PE-conjugated IL-4 (clone 11B11), FITC-conjugated IFNγ (clone XMG1.2), and APC-conjugated CD4 (clone RM4-5) antibodies were purchased from eBioscience. G418 was purchased from Nacalai Tesque. MG132 was purchased from Calbiochem. The MOG_35-55_ peptide (MEVGWYRSPFSRVVHLYRNGK) was synthesized and purified by HPLC (Invitrogen). Pertussis Toxin (PTx) was purchased from List Biological Laboratories. Complete Freund’s adjuvant (CFA) was purchased from Chondrex.

### Cells and transfections

**Figure 1.**
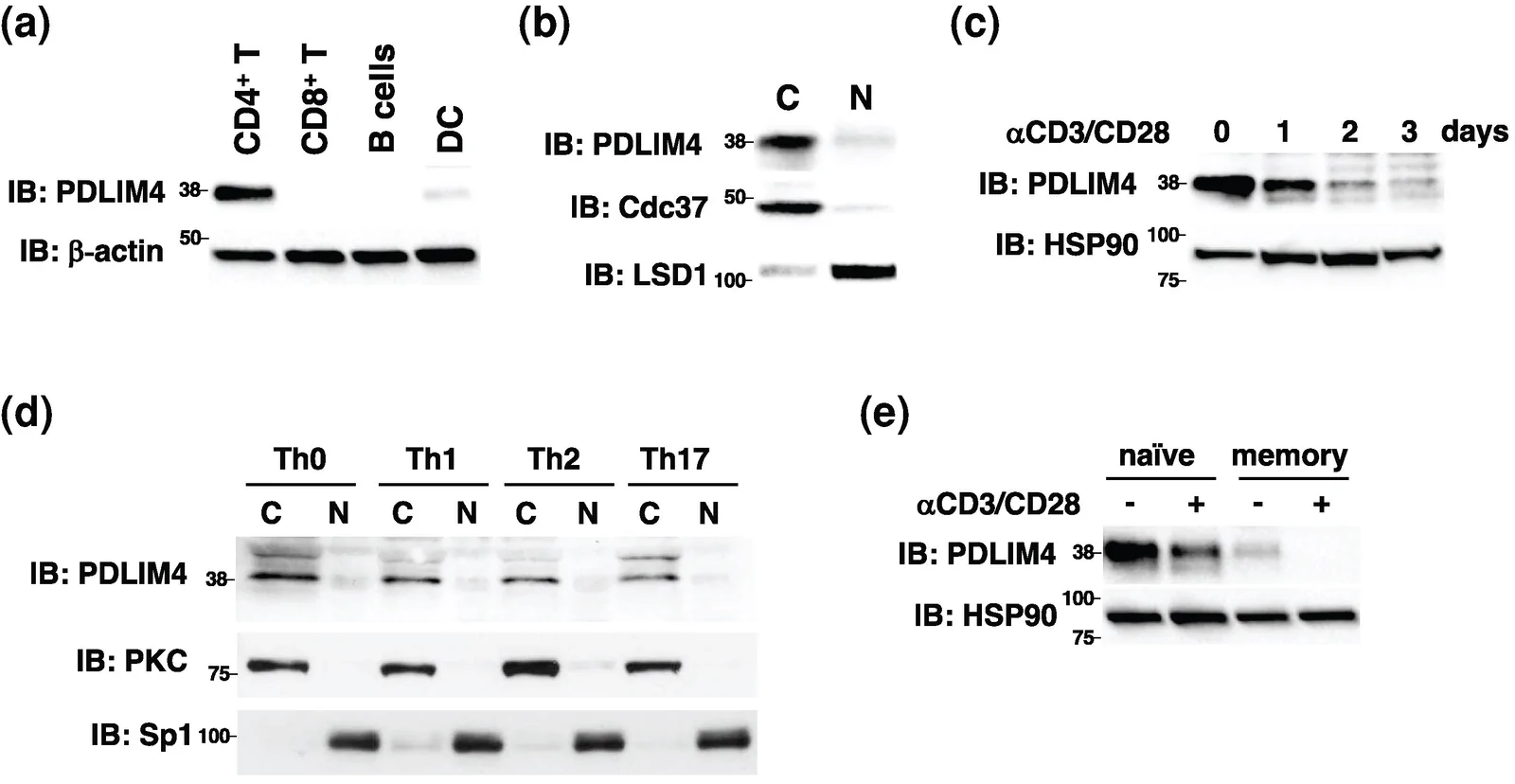
PDLIM4 is a cytoplasmic protein predominantly expressed in CD4^+^ T cells. (a) Expression of PDLIM4 in primary immune cells. Whole cell extracts were subjected to immunoblot (IB) with PDLIM4 and β-actin antibodies. (b) Subcellular localization of PDLIM4 in CD4^+^ T cells. Cytoplasmic (C) and nuclear (N) extracts were subjected to immunoblot with PDLIM4 antibody. The purity of the fractionations was confirmed by blotting with Cdc37 (cytoplasm) and LSD1 (nucleus) antibodies. (c) PDLIM4 expression in activated T cells. CD4^+^ T cells were stimulated without or with CD3 plus CD28 antibodies for 1, 2, and 3 days. Whole cell lysates were analyzed by western blotting with PDLIM4 and HSP90 antibodies. (d) PDLIM4 expression in T-helper cell subsets. Cytoplasmic (C) and nuclear (N) extracts from the indicated subsets of CD4^+^ T cells were subjected to immunoblot with PDLIM4 antibody. The purity of the fractionations was confirmed by blotting with PKC (cytoplasm) and Sp1 (nucleus) antibodies. (e) PDLIM4 expression in naïve and memory CD4^+^ T cells. Purified naïve and memory CD4^+^ T cells were unstimulated or stimulated with CD3 (5 μg/ml) plus CD28 (1 μg/ml) antibodies for 1 day. Whole cell lysates were analyzed by western blotting with PDLIM4 and HSP90 antibodies. Data are representative of five (a), three (b), five (c), four (d), or three (e) independent experiments.

HEK293T cells were purchased from the European Collection of Authenticated Cell Cultures and cultured in DMEM supplemented with 10% FBS. CD4^+^ T, CD8^+^ T, CD19^+^ B cells, and CD11c^+^ dendritic cells were purified from spleen with MACS columns using CD4 (#130-117-043), CD8 (#130-117-128), CD19 (#130-052-201), and CD11c (#130-125-835) MicroBeads, respectively (Miltenyi Biotech). Naïve CD4^+^ T cells were purified from spleen using the CD4^+^CD62^+^ T cell purification kit (#130-106-643; Miltenyi Biotech). CD4^+^ T cells, CD8^+^ T cells, CD19^+^ B cells, and naïve CD4^+^ T cells were cultured in RPMI1640 supplemented with 10% FBS, and CD11c^+^ dendritic cells were cultured in DMEM supplemented with 10% FBS. For the naïve/memory CD4^+^ T cell experiment (Fig. 1e), naïve and memory CD4^+^ T cells were purified from spleen using the EasySep mouse naïve CD4^+^ T Cell isolation kit (ST-19765; Veritas) and the EasySep mouse memory CD4^+^ T Cell isolation kit (ST-19767; Veritas), respectively. For transient transfection, cells were transfected with Effectene (QIAGEN), cultured for 20 hours, and then subjected to the indicated analysis.

### Reporter assays

**Figure 2.**
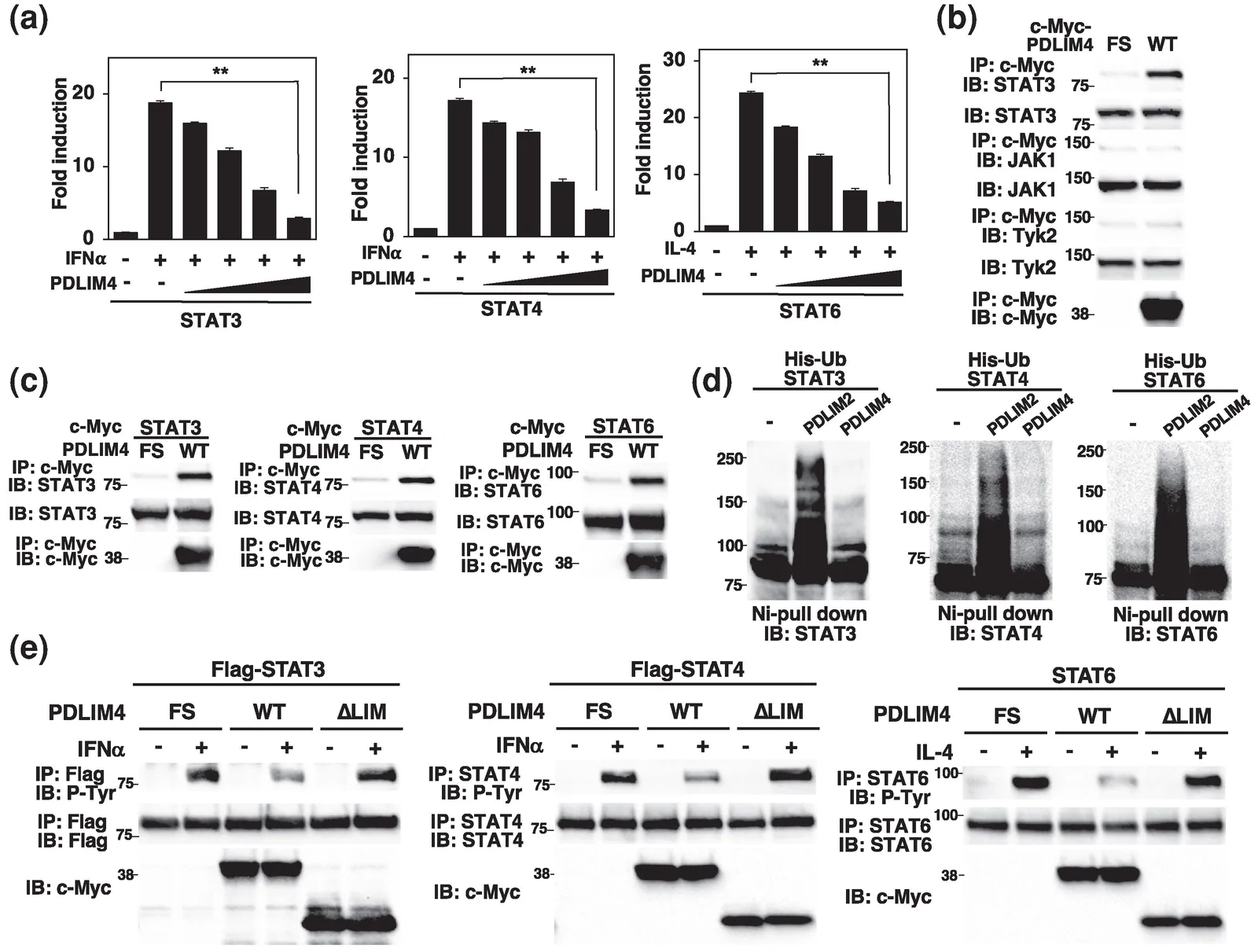
PDLIM4 inhibits STAT3, STAT4, and STAT6-mediated signaling. (a) Luciferase activity in HEK293T cells transfected with the (2x) IRF-1 luciferase reporter plasmid and pRL-Null without or with plasmids encoding STAT3 (left), STAT4 (center), or STAT6 (right) in the absence or presence of increasing amounts (wedge) of PDLIM4 and then stimulated with IFNα (1 ng/ml) or IL-4 (1 ng/ml) for 4 hours. Data are mean ± SD. **P < 0.01. (b) HEK293T cells were transfected with plasmids encoding a c-Myc-tagged PDLIM4 wild-type (WT) or frameshift mutant (FS). Whole cell lysates were immunoprecipitated (IP) with c-Myc antibody and analyzed by western blotting with STAT3, JAK1, or Tyk2 antibodies. (c) HEK293T cells were transfected with plasmids encoding STAT3 (left), STAT4 (center) or STAT6 (right) together with a c-Myc-tagged PDLIM4 wild-type or frameshift mutant. Whole cell lysates were immunoprecipitated with c-Myc antibody and analyzed by western blotting with STAT3 (left), STAT4 (center), or STAT6 (right) antibodies. (d) Ubiquitination assay in HEK293T cells transfected with plasmids encoding histidine-tagged ubiquitin (His-Ub) and STAT3 (left), STAT4 (center), or STAT6 (right), together without or with PDLIM2 or PDLIM4, then treated with MG132 (10 μM) for 2.5 hours. His-tagged proteins were purified by Ni-resin (Ni-pull down). Polyubiquitination of STAT3, STAT4, and STAT6 was detected by STAT3 (left), STAT4 (center), or STAT6 (right) antibodies. (e) HEK293T cells were transfected with plasmids encoding STAT3 (left), STAT4 (center), or STAT6 (right) together with c-Myc-tagged wild-type or the indicated PDLIM4 mutants. Whole cell lysates were immunoprecipitated with Flag (left), STAT4 (center), or STAT6 (right) antibodies, stimulated without or with IFNα (10 ng/ml; left and center) or IL-4 (10 ng/ml; right) for 30 minutes and analyzed by western blotting with a phosphotyrosine antibody. Data are representative of three (a, b, c, d, e) independent experiments.

For the luciferase assay, HEK293T cells were co-transfected with a 2xIRF-1 luciferase reporter or a STAT6-responsive reporter construct and expression plasmids encoding STAT4, STAT6, or STAT3 plus wild-type or mutant PDLIM4. Luciferase activities were measured according to the manufacturer’s instructions (Promega). The Dual-Luciferase Reporter Assay System (Promega) was used in Fig. 2a. In Supplementary Figure S1, STAT3, STAT4, and STAT6-driven luciferase activities were normalized to the expression levels of STAT3, STAT4, or STAT6, which were measured by western blotting.

### Co-immunoprecipitation and immunoblot analysis

All lysis buffers used for western blot analysis contained a proteinase inhibitor cocktail (Roche). Whole cell extracts were prepared by lysing cells in RIPA buffer (50 mM Tris–HCl pH 8.0, 150 mM NaCl, 1% NP-40, 0.5% sodium deoxycholate, 0.1% SDS), buffer consisting of 50 mM Tris pH 8.0, 1% Triton X-100, 0.5 mM EDTA, 150 mM NaCl, and 50 mM sodium fluoride, or buffer consisting of 50 mM Tris 8.0, 0.5% NP-40, 5 mM EDTA, 50 mM NaCl, and 50 mM sodium fluoride. For co-immunoprecipitation experiments, extracts were incubated with the indicated antibodies plus protein-G-Sepharose (GE Healthcare), anti-c-Myc agarose, or DDDDK-tagged Protein PURIFICATION GEL, washed four times with lysis buffer, and analyzed by immunoblot. Cytoplasmic and nuclear extracts were prepared as follows. Cells were first lysed on ice for 3 minutes with hypotonic buffer (20 mM HEPES, pH 8.0, 10 mM KCl, 1 mM MgCl_2_, 0.1% Triton X-100, 20% glycerol). After centrifugation (5000 rpm, 1 minute), supernatants were collected and used as cytoplasmic fractions. The pellets were next lysed by hypertonic buffer (20 mM HEPES, pH 8.0, 1 mM EDTA, 20% glycerol, 0.1% Triton X-100, 400 mM NaCl) for 20 minutes on ice. After centrifugation (15000 rpm, 5 minutes), supernatants were collected and used as nuclear fractions.

### Ubiquitination assay

HEK293T cells were transfected with expression plasmids encoding histidine-tagged ubiquitin together with STAT3, STAT4, or STAT6, and c-Myc-PDLIM2 or PDLIM4 and treated with MG132 (10 μM) for 2.5 hours. His-tagged proteins were purified from whole cell lysates with His60 Nickel Superflow Resin (TAKARA BIO INC.) as previously described (28) and analyzed by immunoblot. Briefly, transfected cells were extracted under denaturing conditions with a buffer containing 6 M guanidium–HCl. Extracts were incubated with His60 Nickel Superflow Resin for 3 hours and then washed with buffer containing 25 mM Tris, pH 6.8, 20 mM imidazole. Purified proteins were subjected to immunoblot analysis.

### Generation of Pdlim4-deficient mice

Murine *pdlim4* genomic DNA was amplified by PCR (KOD plus DNA polymerase, TOYOBO) using C57BL/6 mice genomic DNA. To disrupt *pdlim4*, we constructed a targeting vector in which the neomycin phosphotransferase gene lacking a polyA signal, derived from pMC1 Neo (Stratagene), was inserted into exon 3. The targeting vector was electroporated into Bruce4 ES cells, which originated from C57BL/6 mice. Cells were cultured with G418 (200 μg/ml) for 8 days, and G418-resistant clones were picked. Homologous recombinants were screened by PCR and subsequently confirmed by Southern blot analysis. Targeted ES clones were injected into C57BL/6 blastocysts. Chimeric mice were bred to C57BL/6 mice, and homozygotes were obtained by intercrossing heterozygotes. Mice were maintained under specific pathogen–free conditions. All experiments were in accordance with guidelines approved by the Institutional Animal Care and Use Committee (IACUC) of RIKEN Yokohama Branch.

### In vitro Th differentiation experiments

Naïve CD4^+^ T cells were cultured with plate-bound anti-CD3 (0.2 µg/ml) plus soluble anti-CD28 (1 µg/ml) for 3 days under skewing condition for each Th subsets as follows: IL-12 (2.5 ng/ml) for Th1; IL-4 (10 μg/ml) for Th2; TGF-β (2.5 ng/ml) and IL-6 (10 ng/ml) for Th17. Cells were then restimulated with anti-CD3 (0.2 µg/ml for Th1 and Th2, or 1 µg/ml for Th17) for 20 hours, and the production of IFN-γ, IL-4, and IL-17A in the supernatants was measured by ELISA (BD Bioscience). For PDLIM4 expression, CD4^+^ T cells were cultured with anti-CD3 (1 µg/ml) plus soluble anti-CD28 (1 µg/ml) for 4 days under Th0, 1, 2, and 17 cell-skewing conditions as described above, and cytoplasmic and nuclear fractions were extracted.

### IgE production from B cells

CD19^+^ B cells were purified from spleen and cultured with anti-CD40 (5 μg/ml) plus IL-4 (100 ng/ml) for 5 days. IgE production in the supernatants was measured by ELISA (BD Bioscience).

### Induction of experimental autoimmune encephalomyelitis (EAE)

Mice were immunized subcutaneously with the MOG_35-55_ peptide (100 μg) emulsified in CFA (10 mg/ml) at Day 0. PTx was intraperitoneally injected at Days 0 and 2. The development of neurological signs was monitored daily and graded, with a clinical score from 0 to 5: 0, no sign; 0.5, tail weakness; 1, limp tail; 2, hind limb weakness; 3, hind limb paralysis; 4, hind and fore limb paralysis; 5, moribund state and death. To evaluate the expression of Th1, Th17, and Th2 cytokines, cells were purified from inguinal lymph nodes 13 days after the immunization and cultured without or with MOG_35-55_ peptide (10 μM) for 3 days. The production of IFNγ, IL-17, and IL-4 in the culture supernatant was measured by ELISA (BD Bioscience).

### Nippostrongylus brasiliensis (N. brasiliensis) infection

To evaluate the expulsion of parasites, mice were infected subcutaneously with 800 third-stage larvae of *N. brasiliensis*, and the number of worms in the intestine was counted 6 days after the infection. To determine the percentage of IL-4-producing CD4^+^ T cells, mice were infected subcutaneously with 100 third-stage larvae of *N. brasiliensis*. Cells were purified from mesenteric lymph nodes 7 days after *N. brasiliensis* infection, stimulated with CD3 (plate-bound; 1 μg/ml) plus CD28 (1 μg/ml) antibodies for 7 hours, and subjected to intracellular cytokine staining by flow cytometry using PE-conjugated anti-IL-4, FITC-conjugated anti-IFNγ, and APC-conjugated anti-CD4 as previously described (29). The percentages of IL-4 ^+^ IFNγ^−^ cells in CD4^+^ cells were calculated. To detect IL-4, IL-13, and IFNγ production, mice were infected subcutaneously with 100 third-stage larvae of *N. brasiliensis*. Cells were purified from mesenteric lymph nodes 7 days after *N. brasiliensis* infection and cultured with CD3 (plate-bound; 1 μg/ml) plus CD28 (1 μg/ml) antibodies for 48 hours. IL-4, IL-13, and IFNγ production in the culture supernatant was measured by ELISA (R&D Systems).

### Association analysis of the PDLIM4 nsSNP

Genotyping of the *PDLIM4* nsSNP (rs4877) was performed using TaqMan genotyping assays (Probe ID: C___2965176_10s, Thermo Fisher Scientific), and resulting genotypes were tested with the Cochran-Armitage trend test using the same samples as previously described (30). All samples used for this research were of Japanese origin and were provided by the BioBank Japan Project with written informed consent (31). The association analysis in this study was approved by the ethical committee of the RIKEN Yokohama Institute.

## Results

### PDLIM4 is a cytoplasmic protein mainly expressed in CD4^+^ T cells

We first examined the expression of PDLIM4 in immune cells and found that it was expressed predominantly in CD4^+^ T cells, but not in CD8^+^ T cells, B cells, or dendritic cells (Fig. 1a), although PDLIM1, PDLIM2, and PDLIM7, other LIM proteins, are expressed in all of these immune cells (21–23). This finding prompted us to investigate the role of PDLIM4 in regulating the activation and function of CD4^+^ T cells. We next examined the subcellular localization of PDLIM4 in CD4^+^ T cells and found that it is located predominantly in the cytoplasm, but not in the nucleus (Fig. 1b). We then examined the expression of PDLIM4 before and after activation of CD4^+^ T cells stimulated with CD3 plus CD28 antibodies for 1, 2, and 3 days. PDLIM4 expression was decreased by CD3/CD28 activation, but was still expressed at Day 3 of stimulation (Fig. 1c). We also checked the expression of PDLIM4 after the cells were differentiated into Th1, Th2, or Th17 cells. In contrast to the nuclear expression of PDLIM2 (16), PDLIM4 was expressed in the cytoplasm in all of these T cell subsets (Fig. 1d). We further purified naïve and memory T cells and compared their PDLIM4 expression. The expression of PDLIM4 in memory T cells was markedly reduced compared to that in naïve T cells (Figure 1e).

### PDLIM4 inhibits STAT3-, STAT4-, and STAT6-mediated signaling

As described above, STAT4, STAT6, and STAT3 are critical for the differentiation of naïve CD4^+^ T cells into Th1, Th2, and Th17 cells, respectively. Moreover, we have previously demonstrated that PDLIM2, a LIM protein most structurally similar to PDLIM4, negatively regulates STAT3- and STAT4-mediated signaling (16, 17). We therefore examined the effect of PDLIM4 in STAT4-, STAT6-, and STAT3-mediated activation. We transiently transfected HEK293T cells with a luciferase reporter containing two copies of high-affinity STAT-binding sites from the IRF-1 promoter together with expression plasmids for PDLIM4 and STAT3 or STAT4. Transfection of STAT3 or STAT4 augmented luciferase reporter activity when cells were stimulated with IFNα, while cotransfection of PDLIM4 markedly suppressed IFNα-induced STAT3 and STAT4-mediated transactivation of the reporter in a dose-dependent manner (Fig. 2a, left and center). We further transfected HEK293T cells with a STAT6-responsive luciferase reporter along with PDLIM4 and STAT6. PDLIM4 also suppressed IL-4-induced STAT6-mediated transactivation in a dose-dependent manner (Fig. 2a, right).

Since PDLIM4 is located in the cytoplasm, it might associate with cytoplasmic signaling molecules to suppress cytokine-induced STAT-mediated signal transduction. We therefore transfected HEK293T cells with c-Myc-tagged PDLIM4 and examined if it could interact with endogenous JAK1, Tyk2, or STAT3. PDLIM4 was co-immunoprecipitated with STAT3, but not JAK1 or Tyk2, suggesting that it acts directly on STAT3, but not at the JAK1/Tyk2 level to inhibit cytokine-induced STAT-mediated signaling (Fig. 2b). We then examined whether PDLIM4 associated with STAT3, STAT4, or STAT6 and found that it was co-immunoprecipitated with all these STAT proteins (Fig. 2c), demonstrating that PDLIM4 can interact with STAT3, STAT4, and STAT6 to target them for inactivation.

We next investigated the mechanisms by which PDLIM4 suppressed the activity of STAT proteins. Since PDLIM2 is a ubiquitin E3 ligase that promotes polyubiquitination of STAT3 and STAT4, resulting in their degradation by the proteasome (16, 17), we tested whether PDLIM4 also promotes polyubiquitination of STAT proteins. As shown in Fig. 2d, STAT3, STAT4, and STAT6 were polyubiquitinated when they were coexpressed with PDLIM2. However, PDLIM4 did not polyubiquitinate any of these STAT proteins, suggesting that it does not have ubiquitin E3 ligase activity.

As described above, STAT activity is strictly regulated by its tyrosine phosphorylation. We next examined the effect of PDLIM4 on tyrosine phosphorylation of STAT proteins in HEK293T cells transfected with STAT3, STAT4, or STAT6 without or with PDLIM4. Stimulation of the cells with IFNα or IL-4 induced tyrosine phosphorylation of STAT3 and STAT4 or STAT6, respectively, whereas coexpression of PDLIM4 markedly reduced tyrosine phosphorylation of all these STAT proteins (Fig. 2e). Notably, the PDLIM4 mutant lacking the LIM domain (ΔLIM) was impaired in suppressing tyrosine phosphorylation of all these STAT proteins. We further tested the ability of ΔLIM PDLIM4 to suppress STAT activation by using a luciferase assay and found that ΔLIM PDLIM4 was impaired in suppressing STAT3-, STAT4-, and STAT6-mediated gene activation (Supplementary Figure S1). These data suggest that PDLIM4 inhibits tyrosine phosphorylation and the subsequent activation of STAT proteins in a LIM domain-dependent manner.

### PDLIM4 recruits PTP-BL to dephosphorylate STAT3, STAT4, and STAT6

**Figure 3.**
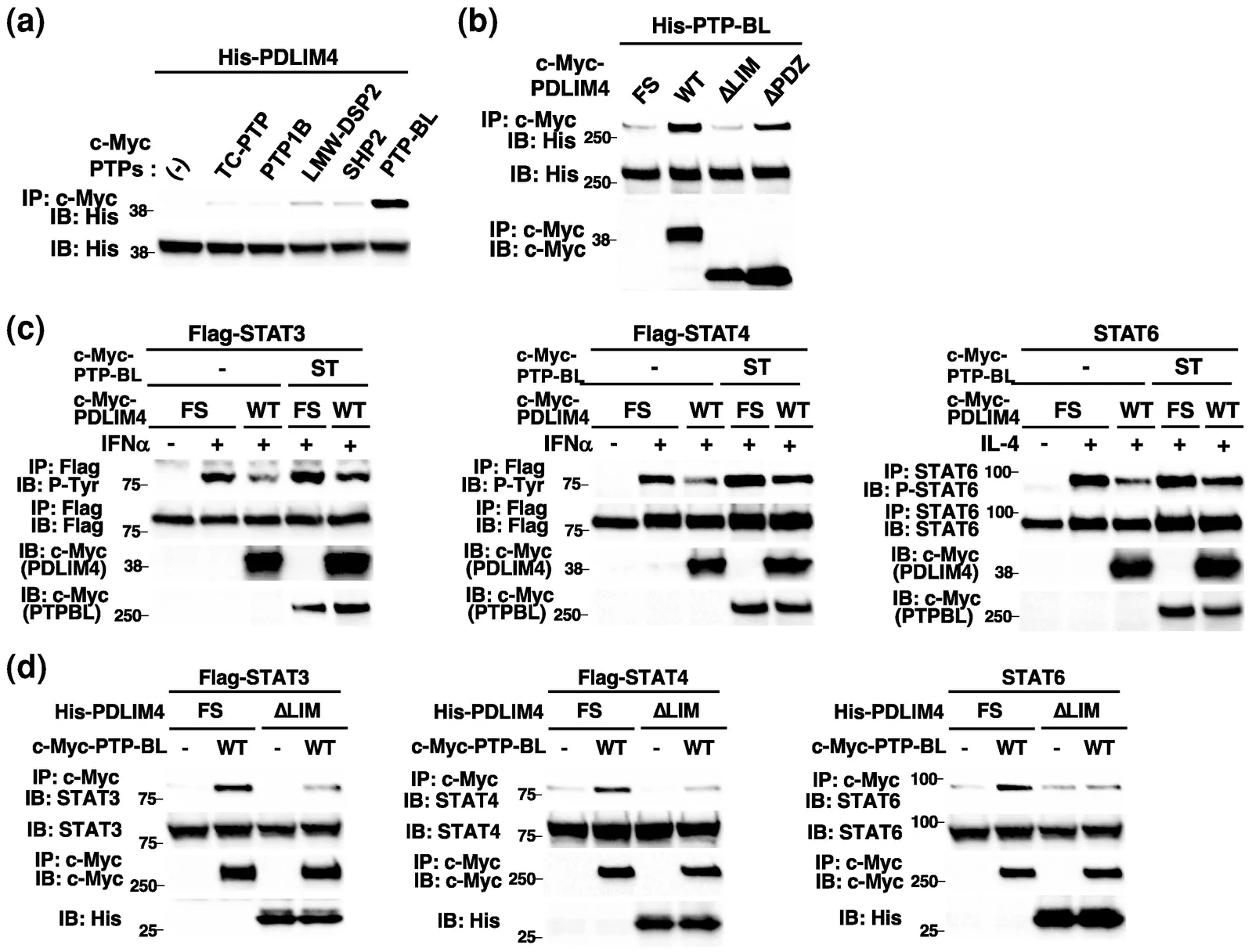
PDLIM4 recruits PTP-BL to dephosphorylate STAT3, STAT4, and STAT6. (a) HEK293T cells were transfected with plasmids encoding His-tagged PDLIM4 together without or with TC-PTP, PTP1B, LMW-DSP2, SHP2, or PTP-BL. Whole cell lysates were immunoprecipitated with a c-Myc antibody and analyzed by western blotting with a His antibody. (b) Critical region of PDLIM4 for interaction with PTP-BL. HEK293T cells were transfected with plasmids encoding His-tagged PTP-BL and c-Myc-tagged wild-type or the indicated PDLIM4 mutants. Whole cell lysates were immunoprecipitated with a c-Myc antibody and analyzed by western blotting with a His antibody. (c) HEK293T cells were transfected with plasmids encoding Flag-tagged STAT3 (left), Flag-tagged STAT4 (center), or untagged STAT6 (right) together without or with c-Myc-tagged PDlIM4 wild-type or frameshift mutant in the absence or presence of the substrate-trapping (ST) PTP-BL mutant, and stimulated without or with IFNα (10 ng/ml; left and center) or IL-4 (10 ng/ml; right) for 30 minutes. Whole cell lysates were immunoprecipitated with Flag (left and center) or STAT6 (right) antibodies and analyzed by western blotting with phosphotyrosine (left and center) or phospho-STAT6 (right) antibodies. (d) HEK293T cells were transfected with plasmids encoding STAT3 (left), STAT4 (center), or STAT6 (right) together without or with c-Myc-tagged PTP-BL in the absence or presence of the ΔLIM PDLIM4 mutant. Whole cell lysates were immunoprecipitated with anti-c-Myc antibody and analyzed by western blotting with STAT3 (left), STAT4 (center), or STAT6 (right) antibodies. Data are representative of four (a) or three (b, c, d) independent experiments.

PDLIM4 itself is known to lack phosphatase activity. Since both PDZ and LIM domains are involved in protein–protein interactions, we predicted that PDLIM4 might interact with PTPs via either of these domains to suppress STAT phosphorylation. To identify PTPs that can bind to PDLIM4 and promote dephosphorylation of STAT proteins, we transfected HEK293T cells with plasmids encoding His-tagged PDLIM4 and c-Myc tagged TC-PTP, PTP1B, LMW-DSP2, SHP2, or PTP-BL, PTPs that were previously reported to dephosphorylate any of the STAT molecules (5–10, 12, 13). The expression of these PTPs was confirmed by immunoblot with a c-Myc antibody (Supplementary Figure S2). Among these PTPs, PTP-BL was predominantly co-immunoprecipitated with PDLIM4 (Fig. 3a). We then determined which domain of PDLIM4 was essential for the association with PTP-BL. A ΔLIM PDLIM4 mutant failed to interact with PTP-BL, while a PDLIM4 mutant lacking the PDZ domain (ΔPDZ) could still bind to PTP-BL (Fig. 3b), suggesting that PDLIM4 binds to PTP-BL via its LIM domain, which is consistent with previous reports showing that the PDZ domains of PTP-BL can bind to the LIM domain of PDLIM4 (32, 33). This finding is consistent with the results shown in Fig. 2d that PDLIM4 suppresses STAT phosphorylation in a LIM domain-dependent manner. We next investigated whether PTP-BL is involved in PDLIM4-mediated tyrosine dephosphorylation of STAT proteins by using a substrate-trapping (ST) mutant of PTP-BL, in which the catalytic cysteine residue in the phosphatase domain was substituted with serine. The ST mutants often function as dominant negatives to inactivate a specific protein tyrosine phosphatase *in vivo*, since they bind to the substrate but lack the phosphatase activity (34, 35). We therefore transfected HEK293T cells with STAT3, STAT4, or STAT6 and PDLIM4 together with or without the ST mutant of PTP-BL (ST-PTP-BL). PDLIM4 suppressed IFNα-induced tyrosine phosphorylation of STAT3 and STAT4 and IL-4-induced tyrosine phosphorylation of STAT6, whereas ST-PTP-BL reverted the decreased tyrosine phosphorylation of all these STAT proteins, suggesting that PTP-BL is responsible for PDLIM4 inhibition of tyrosine phosphorylation of these STAT proteins (Fig. 3c). These results prompted us to test the possibility that PDLIM4 functions as an adaptor recruiting PTP-BL to dephosphorylate STAT proteins. Since the ΔLIM PDLIM4 mutant failed to bind to PTP-BL but still bound to STAT3, STAT4, and STAT6 (Supplementary Figure S3), overexpression of ΔLIM PDLIM4 in cells should act as a dominant negative to interfere with PDLIM4-mediated binding of STAT proteins to PTP-BL. We therefore examined the interaction of STAT3, STAT4, or STAT6 with PTP-BL in the absence or presence of the ΔLIM PDLIM4 mutant. Overexpression of ΔLIM PDLIM4 impaired the binding of PTP-BL to all these STAT proteins (Fig. 3d), indicating that PDLIM4 could mediate their binding to PTP-BL. We next examined the specificity of these functions of PDLIM4 for PTP-BL. Since STAT3 and STAT6 also bind to TC-PTP and PTP1B and thus could be dephosphorylated by these PTPs (6–9), we transfected HEK293T cells with STAT3 or STAT6 and TC-PTP or PTP1B together with or without the ΔLIM PDLIM4 mutant. The ΔLIM PDLIM4 mutant did not affect the interaction of either STAT3 with TC-PTP and PTP1B or STAT6 with TC-PTP and PTP1B (Supplementary Figure S4), which is consistent with the finding of selective binding of PDLIM4 to PTP-BL (Fig. 3a). These data demonstrated that PDLIM4 functions as an adaptor to specifically recruit PTP-BL to STAT proteins through its LIM domain and promote their dephosphorylation by PTP-BL, thereby suppressing STAT-mediated signaling.

### Enhanced Th1, Th2, and Th17 cell differentiation in Pdlim4^−/−^ mice

**Figure 4.**
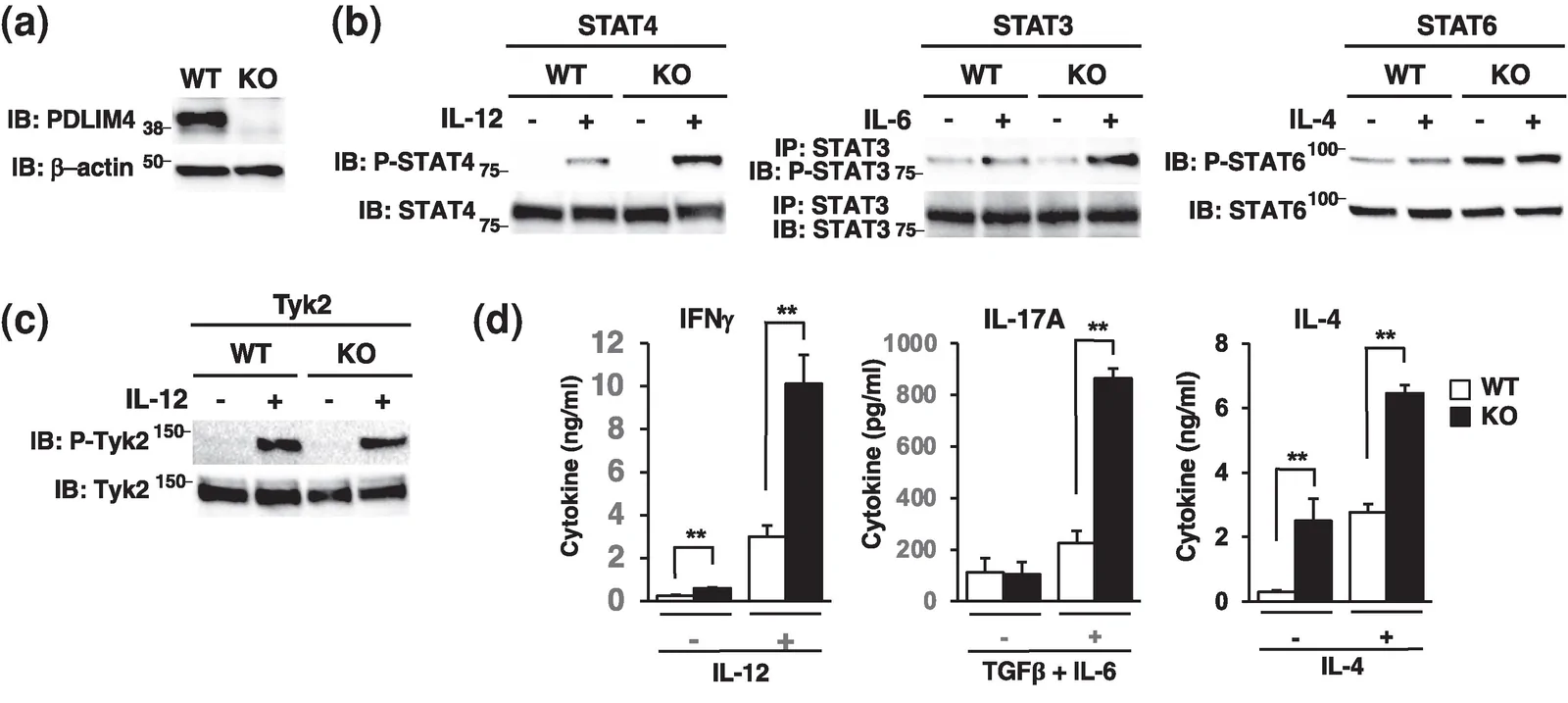
Enhanced *in vivo* Th1, Th2, and Th17 cell differentiation in *Pdlim4^−/−^* mice. (a) Western blot analysis of PDLIM4 expression in whole cell lysates of naïve CD4^+^ T cells from wild-type (WT) and PDLIM4-deficient mice (KO). (b) CD4^+^ T cells (left and center) or naive CD4^+^ T cells (right) were cultured with anti-CD3 plus anti-CD28 for 3 days and stimulated with IL-12 (left), IL-6 (center), or IL-4 (right) for 30 minutes. Whole cell lysates were subjected to immunoprecipitation with a STAT3 antibody and immunoblot with anti-phospho-STAT3 (center), or analyzed by western blotting with anti-phospho-STAT4 (left) or anti-phospho-STAT6 (right). (c) CD4^+^ T cells were cultured with anti-CD3 plus anti-CD28 for 3 days and stimulated with IL-12 for 30 minutes. Whole cell lysates were analyzed by western blotting with anti-phospho-Tyk2. (d) Naïve CD4^+^ T cells purified from wild-type and PDLIM4-deficient mice were differentiated under unskewed (Th0), Th1-, Th2-, or Th17-skewing conditions for 4 days and restimulated with anti-CD3. The production of IFNγ (left), IL-17A (center), and IL-4 (right) was measured by ELISA. (mean ± SD; **P < 0.01). Data are representative of four (a, b, c) or three (d) independent experiments.

To investigate the role of PDLIM4 *in vivo*, we generated *Pdlim4*-deficient mice by gene targeting. Western blot analysis of CD4^+^ T cells revealed that PDLIM4 expression was not detected in the knockout mice (Fig. 4a). *Pdlim4^−/−^* mice have normal numbers of splenic immune cells, including CD4^+^ T cells, CD8^+^ T cells, B cells, macrophages, and dendritic cells as compared to *Pdlim4^+/+^* mice (data not shown). Since PDLIM4 facilitates the dephosphorylation of STAT3, STAT4, and STAT6, as shown in Fig. 2e, we first examined the phosphorylation status of these STAT proteins in *Pdlim4*-deficient CD4^+^ T cells. The levels of tyrosine phosphorylation of STAT3, STAT4 and STAT6 in CD4^+^ T cells in response to IL-6, IL-12, or IL-4, respectively, were markedly enhanced in *Pdlim4*-deficient CD4^+^ T cells as compared to wild-type mice (Fig. 4b). In contrast, IL-12-induced tyrosine phosphorylation of Tyk2 was comparable between *Pdlim4^+/+^* and *Pdlim4^−/−^* CD4^+^ T cells (Fig. 4c). These data are consistent with our above data showing that PDLIM4 selectively binds to STAT proteins but not JAK family kinases (Fig. 2b and c). Since STAT4-, STAT6-, and STAT3-mediated signaling are essential for Th1, Th2, and Th17 cell differentiation, respectively, we then examined the effect of PDLIM4 deficiency on the development of these Th-cell subsets. Naïve CD4^+^ T cells were purified from the spleen of wild-type and *Pdlim4*-deficient mice and cultured under Th1-, Th2-, and Th17-skewing conditions with anti-CD3 and IL-12, IL-4, or TGFβ plus IL-6, respectively. Compared to *Pdlim4^+/+^* CD4^+^ T cells, *Pdlim4^−/−^* CD4^+^ T cells had a striking increase in IFNγ, IL-4 and IL-17A production in Th1, Th2, or Th17 cells, respectively (Fig. 4d). These data again indicate that PDLIM4 acts directly on STAT4, STAT6, and STAT3 to inhibit their tyrosine phosphorylation and subsequent Th1, Th2, and Th17 cell differentiation, but not at the level of TyK2. On the other hand, the production of IgE from B cells was comparable between *Pdlim4^+/+^* and *Pdlim4^−/−^* CD4^+^ T mice (Supplementary Figure S5), which is consistent with the lack of PDLIM4 expression in B cells.

### Enhanced *in vivo* Th1-, Th2-, and Th17-type immune responses in Pdlim4^−/−^ mice

**Figure 5.**
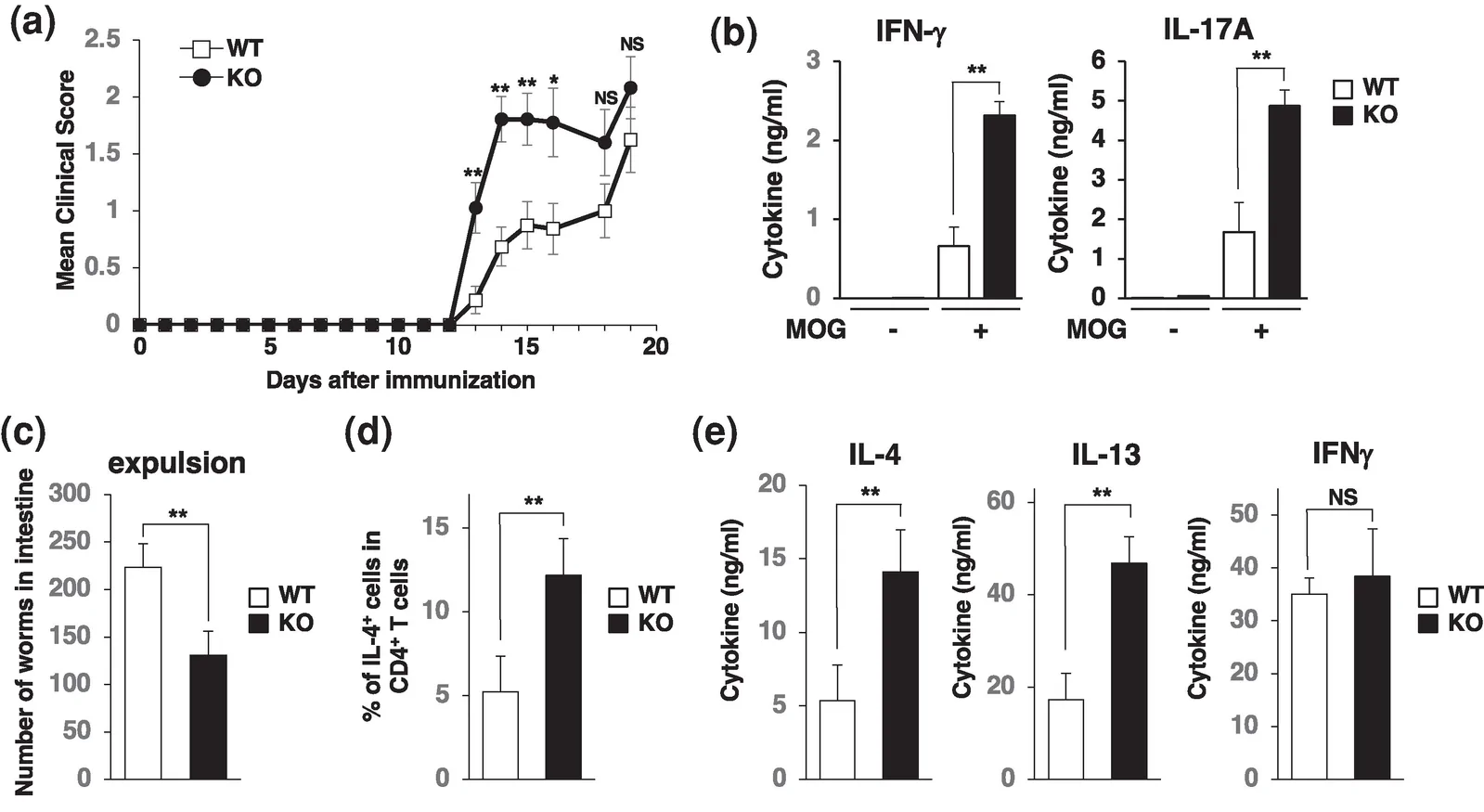
Enhanced *in vivo* Th1-, Th2-, and Th17-type immune responses in *Pdlim4^−/−^* mice. (a) Clinical scores of EAE in wild-type (WT; *n* = 16) and PDLIM4-deficient mice (KO; *n* = 18), pooled from three independent experiments. Data are mean ± SEM; *P < 0.05, **P < 0.01, NS: not significant. (b) IFNγ and IL-17A production in lymph node cells from wild-type (*n* = 3) and PDLIM4-deficient mice (*n* = 3), 13 days after the immunization without or with MOG. Cells were restimulated with MOG peptide (10 μM) for 3 days, and the production of IFNγ and IL-17 in the culture supernatant was measured by ELISA. Data are mean ± SD; **P < 0.01. (c) The number of *N. brasiliensis* remaining in the intestine of wild-type (*n* = 10) and PDLIM4-deficient mice (*n* = 10) 6 days after infection. Data are representative of three independent experiments (mean ± SEM; **P < 0.01). (d) The percentage of IL-4-producing CD4^+^ T cells in the mesenteric lymph node of wild-type (*n* = 4) and PDLIM4-deficient mice (*n* = 4) 7 days after *N. brasiliensis* infection was determined by intracellular cytokine staining with flow cytometry. Data are mean ± SD; **P < 0.01. (e) IL-4 and IL-13 production in lymph node cells from wild-type (*n* = 4) and PDLIM4-deficient mice (*n* = 4) 7 days after *N. brasiliensis* infection. Cells were restimulated with anti-CD3 plus anti-CD28 for 2 days, and the production of IL-4, IL-13, and IFNγ in the culture supernatant was measured by ELISA. Data are mean ± SD; **P < 0.01.

To examine the effect of PDLIM4 deficiency on *in vivo* Th1 and Th17 responses, we immunized *Pdlim4^+/+^* and *Pdlim4^−/−^* mice with MOG_33-55_ peptide to induce EAE, in which both Th1 and Th17 cells are responsible for the pathogenesis (36). The neurological signs began to be observed in both mice from Day 13 after the immunization, but the severity of disease in *Pdlim4^−/−^* mice was significantly higher than that in *Pdlim4^+/+^* mice (Fig. 5a). We then examined the production of Th1- and Th17-type cytokines in cells from the inguinal lymph nodes of immunized mice. Cells from *Pdlim4^−/−^* mice produced significantly higher amounts of IFNγ and IL-17A following restimulation with MOG peptide *in vitro* than those from *Pdlim4^+/+^* mice (Fig. 5b). Meanwhile, the production of IL-4 could not be detected in cells from both *Pdlim4^+/+^* and *Pdlim4^−/−^* mice. These data suggest that the *in vivo* differentiation of Th1 and Th17 cells was enhanced by PDLIM4-deficiency.

To investigate the effect of PDLIM4 deficiency on an *in vivo* Th2 response, we infected *Pdlim4^+/+^* and *Pdlim4^−/−^* mice with *N. brasiliensis*, an intestinal parasitic nematode that selectively induces Th2-type responses (37), and examined the expulsion of the parasite from the intestine. Six days after infection, *Pdlim4^−/−^* mice have significantly lower numbers of worms in the small intestine compared to *Pdlim4^+/+^* mice (Fig. 5c), indicating that the ability to eliminate the parasites from the intestine, which is an *in vivo* Th2 cell-dependent response, was facilitated in *Pdlim4^−/−^* mice. Consistently, CD4^+^ T cells from infected *Pdlim4^−/−^* mice revealed a higher percentage of IL-4-producing cells (Fig. 5d) and produced greater amounts of IL-4 and IL-13, but not IFNγ, following restimulation with anti-CD3 *in vitro* than *Pdlim4^+/+^* mice (Fig. 5e). These data suggest that *in vivo* differentiation of Th2 cells was enhanced in *Pdlim4^−/−^* mice. Taken together, in Th1/Th17-dominant inflammation, PDLIM4 regulates IFNγ and IL-17, but not IL-4, whereas in Th2-dominant immunity, PDLIM4 regulates only IL-4, not IFNγ or IL-17.

### A G259C PDLIM4 variant was impaired in binding to PTP-BL and suppressing STAT3 activation, and is associated with the risk of rheumatoid arthritis

**Figure 6.**
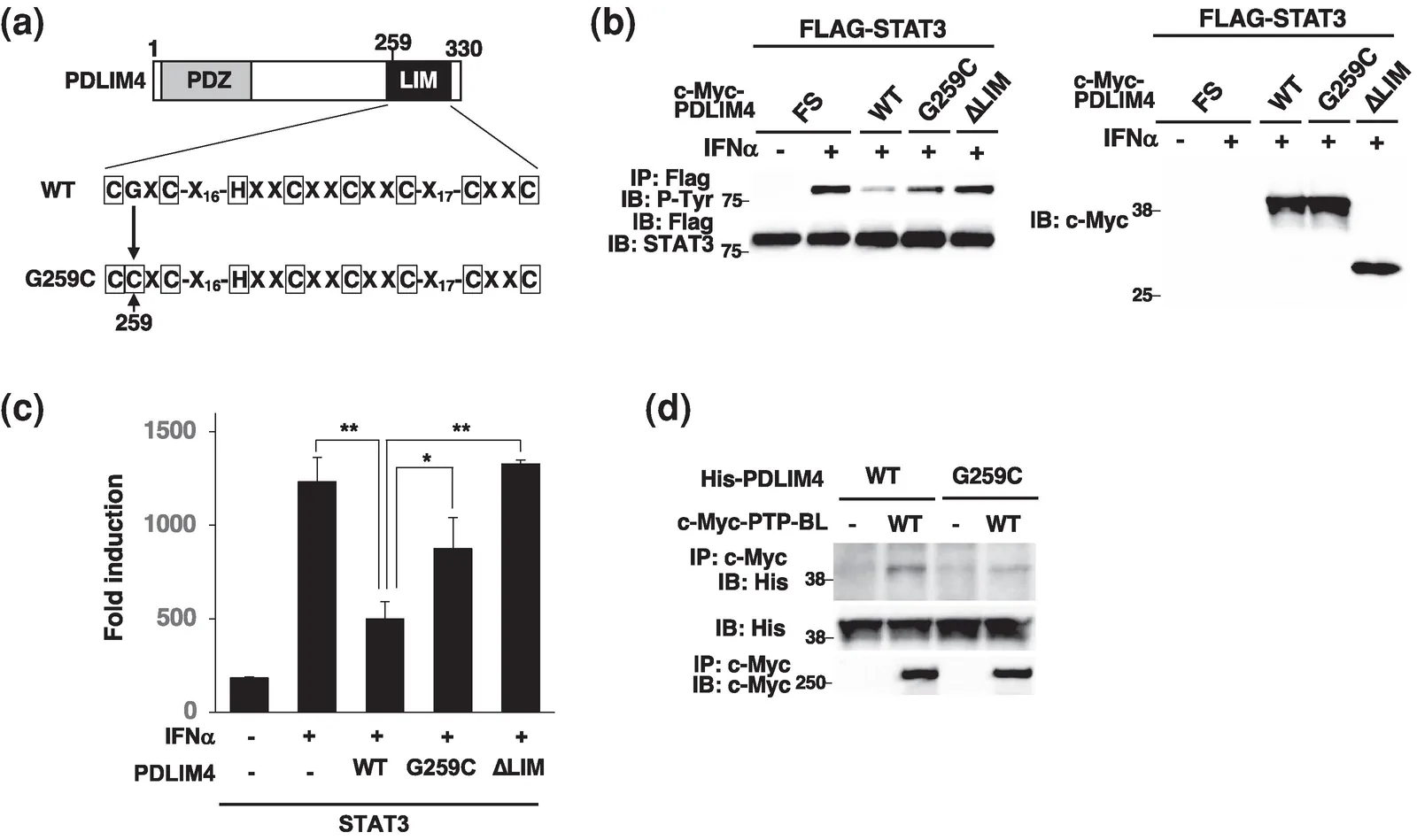
The G259C PDLIM4 mutant is impaired in binding to PTP-BL and suppressing STAT3 activation. (a) The schematic diagram of rs4877, an nsSNP of the *PDLIM4* gene. Numbers show the amino acid position. In this variant, the 259-glycine next to the first conserved cysteine in the LIM domain was replaced with a cysteine, resulting in an added cysteine residue in addition to the normal eight conserved cysteines/histidines, which are boxed, in this domain. (b) HEK293T cells were transfected with plasmids encoding Flag-tagged STAT3 together without or with c-Myc-tagged wild-type or the indicated mutants of PDLIM4 and stimulated without or with IFNα (10 ng/ml) for 30 min. Whole cell lysates were immunoprecipitated with a Flag antibody and analyzed by western blotting with a phosphotyrosine antibody. (c) Luciferase activity in HEK293T cells transfected with the (2x) IRF-1 luciferase reporter plasmid and pRL-Null without or with plasmids encoding STAT3 without or with c-Myc-tagged wild-type or the indicated mutants of PDLIM4 and stimulated with IFNα (1 ng/ml) for 4 hours. Data are mean ± SD. *P < 0.05, **P < 0.01. (d) HEK293T cells were transfected with plasmids encoding His-tagged wild-type or the G259C mutants of PDLIM4 together without or with c-Myc-tagged PTP-BL. Whole cell lysates were immunoprecipitated with a c-Myc antibody and analyzed by western blotting with a His antibody. Data are representative of five (b, c, d) or three (d) independent experiments.

We finally investigated whether dysfunctions of the human *PDLIM4* gene were related to the pathogenesis of human autoimmune diseases. SNPs are genetic variations caused by single-base substitutions that occur at a frequency of more than 1% in the population. Although most SNPs have little or no effect on human development or health, some SNPs affect susceptibility to diseases. We therefore attempted to identify SNPs in the human *PDLIM4* gene that were associated with autoimmune diseases. We searched the “1000 Genome Project” database (38) and selected nsSNP; rs4877, in which 775-guanine in the coding region was replaced by thymine, resulting in an amino acid substitution of 259-glycine to cysteine (G259C) in the LIM domain (Fig. 6a). As described above, a LIM domain contains eight conserved cysteine and histidine residues, which are important for forming the tertiary structure of this domain. The G259C substitution adds an additional cysteine residue, which may affect the structure and function of the LIM domain (19). We assessed the association of rs4877 with rheumatoid arthritis and Graves’ disease by genotyping 2295 rheumatoid arthritis cases, 1769 Graves’ disease cases, and 3401 control subjects, respectively. We found that rs4877 is significantly associated with susceptibility to both rheumatoid arthritis and Graves’ disease, with odds ratios of 1.13 and 1.16, respectively (Table 1). It is well known that the excessive activation of Th17 cells contributes to the pathogenesis of both of these diseases (39), so that the dysfunction of PDLIM4 might be involved in Th17 cell-driven human autoimmune diseases.

**Table 1.** Association analysis of rs4877 in *PDLIM4* with rheumatoid arthritis and Graves’ disease rs4877 (Cys259Gly).

Disease	Sum		Risk allele frequency		Odds ratio (95% CI)	*P*
	Case	Control	Case	Control	Allelic comparison	Cochran Armitage’s trend test
RA	2295	3401	0.318	0.293	1.13 (1.4–1.22)	0.0041
Graves’	1769	3401	0.324	0.293	1.16 (1.06–1.26)	0.00099

We next clarified the effect of this amino acid substitution in rs4877 on PDLIM4 protein function. Since a LIM domain is required for PDLIM4 to recruit PTP-BL and suppress STAT3 phosphorylation and subsequent activation, we hypothesized that the insertion of additional cysteine residues into the LIM domain might impair these PDLIM4 activities, resulting in enhanced Th17-cell differentiation. To test this hypothesis, we generated a c-Myc-tagged PDLIM4 expression construct containing the G259C mutation. We then examined the activity of the G259C PDLIM4 mutant to suppress STAT3 activation. As also seen in Fig. 2a and e, wild-type PDLIM4 inhibited IFNα-induced STAT3 phosphorylation and STAT3-mediated reporter activation, whereas the ΔLIM PDLIM4 mutant failed to inhibit these STAT3 activities (Fig. 6b and c). Notably, the G259C PDLIM4 mutant partially impaired the ability to suppress STAT3 phosphorylation and STAT3-mediated gene activation, indicating that this mutation could affect the function of PDLIM4. Since the LIM domain of PDLIM4 is essential for its binding to PTP-BL, we next tested the interaction of the G259C PDLIM4 mutant with PTP-BL and found that its association with PTP-BL was substantially reduced compared to that of wild-type PDLIM4 (Fig. 6d). These data suggest that PDLIM4 with the G259 mutation could not fully suppress STAT3 activation because ofo insufficient binding to PTP-BL, leading to the overactivation of Th17 cells, which is associated with an increased risk of autoimmune diseases. Thus, the dysfunction of PDLIM4 possibly leads to disease onset and/or development.

## Discussion

In this study, we identify PDLIM4 as a novel regulator for CD4^+^ T cells that suppresses their activation and differentiation into Th subsets. Among immune cells, PDLIM4 is selectively expressed in CD4^+^ T cells. PDLIM4 binds to STAT3, STAT4, and STAT6 and inhibits gene activation mediated by these STAT proteins. Mechanistically, PDLIM4 also binds to and recruits the protein tyrosine phosphatase PTP-BL through its LIM domain, which dephosphorylates tyrosine residues essential for the activation of STAT3, STAT4, and STAT6, thereby inactivating these STAT proteins. Consistently, in PDLIM4-deficient CD4^+^ T cells, tyrosine phosphorylation of STAT3, STAT4, and STAT6, and *in vitro* Th1, Th2, and Th17 cell differentiation were enhanced compared to wild-type cells. Moreover, we also demonstrated that *in vivo* Th1-, Th2-, and Th17-type responses are augmented in PDLIM4-deficient mice compared to wild-type mice using EAE, a Th1 and Th17 cell-mediated mouse inflammation model, and *N. brasiliensis* infection, which induces an *in vivo* Th2 response. These data indicated that PDLIM4 negatively regulates the differentiation of naïve CD4^+^ T cells into Th1, Th2, and Th17 cells by inactivating STAT4, STAT6 and STAT3 proteins.

We previously reported that PDLIM2, another LIM protein, binds to STAT4 and STAT3 and negatively regulates Th1 and Th17 cell differentiation. We demonstrated that the LIM domain of PDLIM2 has ubiquitin E3 ligase activity. PDLIM2 acts as a ubiquitin E3 ligase that promotes the polyubiquitination and proteasome-dependent degradation of STAT4 and STAT3 through its LIM domain, thereby inactivating these STAT proteins. Protein ubiquitination requires three enzymes: a ubiquitin-activating enzyme (E1), a ubiquitin-conjugating enzyme (E2), and a ubiquitin ligase (E3). In the ubiquitination reaction, ubiquitin E3 ligases bind to both target proteins and E2 and then transfer ubiquitin molecules from E2 to the target protein. Since the LIM domain is thought to be a protein-protein interaction domain, PDLIM2 binds to the E2 enzyme through its LIM domain, recruits it to STAT4 and STAT3, and then mediates the polyubiquitination of these STAT proteins. On the other hand, PDLIM4 cannot bind to E2, but it does have the ability to bind to protein tyrosine phosphatases and recruit them to STAT molecules, thereby dephosphorylating and inactivating them. Although eight regularly spaced cysteine (or histidine) residues in the LIM domain are well conserved, the regions of the LIM domain other than these eight conserved cysteines (or histidines) are relatively less homologous among LIM proteins. The structural differences of these regions in the LIM domain confer the diversity of target proteins to which each LIM domain binds. Notably, PDLIM2 and PDLIM7 act as a ubiquitin E3 ligase for the p65 subunit of NF-κB, promoting its polyubiquitination and proteasome-dependent degradation (21, 22), whereas PDLIM1 does not have ubiquitin E3 ligase activity but sequestrates the p65 subunit of NF-κB in the cytoplasm by interaction with α-actinin, an actin-binding protein, via its PDZ domain, thereby inactivating NF-κB signaling (23). These data indicate that PDZ-LIM proteins function as adaptor proteins that bind to and recruit different intracellular factors via their LIM domains (and also probably via their PDZ domains) and inactivate target proteins through different mechanisms, thereby negatively regulating various signaling pathways. We speculate that LIM proteins are a novel family of negative regulators for the immune system.

Previous studies identified three other adaptor proteins that enhance the dephosphorylation of STAT proteins, thereby suppressing their gene activation function. β-Arrestin1 recruits TC-PTP to STAT1, whereas GdX (X-linked gene in the G6PD cluster at Xq28)/UBL4A (Ubiquitin-like protein 4A) and SIPAR (STAT3-interacting protein as a repressor) recruit TC-PTP to STAT3 (40–42). All of these adaptor proteins regulate STAT dephosphorylation only through TC-PTP, but not other PTPs, since their activities to dephosphorylate STAT1 or STAT3 are completely dependent on the expression of TC-PTP. We showed that PDLIM4 selectively recruits PTP-BL, but not other PTPs, such as TC-PTP, PTP1B, LMW-DSP2, or SHP2. We speculate that these adaptor proteins determine the specificity of which phosphatases act on each STAT protein, depending on the cell type or signaling pathways. PDLIM4 could be the CD4^+^ T cell-specific adaptor protein for the regulation of STAT signaling.

Since PDLIM4 is mainly localized in the cytoplasm, it is thought to dephosphorylate STAT proteins in the cytoplasm before they translocate into the nucleus. PDLIM4 is therefore involved in regulating the intensity but not the duration of tyrosine phosphorylation. In naïve, unstimulated CD4^+^ T cells, PDLIM4 is highly expressed and binds to and inhibits the phosphorylation of STAT proteins, thereby maintaining tyrosine phosphorylation of STAT proteins at low levels even with cytokine stimulation. In contrast, upon activation of CD4^+^ T cells by anti-CD3/CD28 (antigen-equivalent) stimulation, PDLIM4 expression is downregulated, and STAT proteins would then be released and highly phosphorylated. Moreover, PDLIM4 expression is very low in memory CD4^+^ T cells even without stimulation, which enables memory CD4^+^ T cells to be readily activated. This may contribute to the rapid and enhanced activation of memory CD4^+^ T cells in response to stimulation.

Notably, IFNγ and IL-17A production from PDLIM4-deficient cells was markedly increased compared to that from wild-type cells, primarily under Th1- and Th17-skewing conditions, respectively, whereas IL-4 production from PDLIM4-deficient cells was significantly enhanced not only under Th2-skewing conditions but also under unskewed conditions (Fig. 4d). There are two possibilities to explain this difference. First, activated T cells produce IL-4 even under unskewed conditions, so that STAT6 can be phosphorylated in unskewed cells without exogenous IL-4 (Fig. 4b). Since PDLIM4 also suppresses phosphorylation of STAT6 induced by endogenous IL-4, PDLIM4-deficient cells showed enhanced IL-4 production compared to wild-type cells even without exogenous IL-4 stimulation.

The second possibility is that PDLIM4 may also control the STAT6-mediated Th2 response through an additional mechanism, which is different from those that control the STAT4-mediated Th1 response and the STAT3-mediated Th17 response. We have shown that PDLIM4 suppressed STAT4-, STAT3-, and STAT6-mediated gene activation in a LIM domain-dependent manner (Supplementary Figure S1). Notably, the ΔLIM mutant of PDLIM4 is almost completely impaired in its ability to suppress STAT4 and STAT3 activation, whereas the activity of ΔLIM PDLIM4 to suppress STAT6 was only partially impaired, suggesting that PDLIM4 may regulate STAT6 activity not only by recruiting PTP-BL through LIM domain, but also by the LIM domain/PTP-BL-independent mechanism. This may account for the enhanced IL-4 production in PDLIM4-deficient cells even under unskewed conditions. However, further studies are required to clarify this different mechanism, which acts only when PDLIM4 suppresses STAT6 activity.

The STAT family of transcription factors is involved in the pathogenesis of various human autoimmune and inflammatory diseases, as shown by genome-wide association studies (GWAS), which identify the association of genetic variation, mostly SNPs, and disease susceptibility. For example, STAT3 polymorphisms are associated with Crohn’s disease and multiple sclerosis (43). STAT4 SNPs are associated with rheumatoid arthritis, systemic lupus erythematosus, systemic sclerosis, and Sjogren’s syndrome (44), while STAT6 SNPs increase the risk of atopic dermatitis and asthma (45). Moreover, these findings were reinforced by the recent identification of gain-of-function (GOF) mutants of STAT proteins. STAT3 GOF variants lead to early-onset lymphoproliferation and autoimmunity (46). STAT4 GOF variants are presented with a severe childhood-onset systemic inflammatory disorder (47), while STAT6 GOF is characterized by severe early-onset multiorgan allergies, including atopic dermatitis (48). In this study, we demonstrated that the nsSNP; rs4877, in the LIM domain of the human *PDLIM4* gene is associated with the risk of rheumatoid arthritis and Graves’ disease. The LIM domain consists of 55 amino acids and forms a double zinc finger structure (19). The LIM domain contains eight conserved cysteine (or histidine) residues, which serve as zinc-binding residues. The first, second, third, and fourth cysteines coordinate the first zinc ion, and the fifth, sixth, seventh, and eighth cysteines coordinate the second zinc ion. The formation of tandem zinc finger structures by eight conserved zinc-binding residues is essential for the LIM domain to function as a protein-binding domain. In nsSNP; rs4877, the glycine immediately adjacent to the first cysteine is replaced with a cysteine. This variation therefore confers an additional cysteine residue to the eight conserved cysteine/histidine residues, which may affect the zinc-binding ability and the tertiary structure formation of the LIM domain. In fact, PDLIM4 with this amino acid substitution partially impaired its ability to bind to PTP-BL and dephosphorylate STAT3, indicating that the insertion of an additional cysteine residue into the LIM domain may cause PDLIM4 dysfunction, promote Th17 cell differentiation, and increase the susceptibility to Th17-driven autoimmune diseases. Considering that Th1- and Th2-type responses, as well as the Th17-type response, are also enhanced in PDLIM4-deficient mice, the nsSNP of PDLIM4 may associate with the risk of Th1 and Th2 cell-driven autoimmune and inflammatory diseases, although further studies will be required to confirm this.

Taking these findings together, we conclude that PDLIM4 is one of the causal genes of Th17-driven autoimmune diseases, including rheumatoid arthritis and Graves’ disease. The PDLIM4-mediated pathway represents a useful new molecular target for the treatment of Th17 cell (and possibly also Th1 and Th2 cell)-mediated human autoimmune and inflammatory diseases.

## Supplementary Material

dxag019_Supplementary_Data
