# Supplementary material for: PDLIM4 promotes dephosphorylation of STAT transcription factors by recruiting PTP-BL and inhibits Th1, Th2, and Th17 cell differentiation
Source: Int Immunol. 2026 May 25;38(9):555–68. doi: 10.1093/intimm/dxag019 (PMC13529576; doi:10.1093/intimm/dxag019)
Supplement: Supplementary file 1 [file dxag019_supplementary_data.pdf]

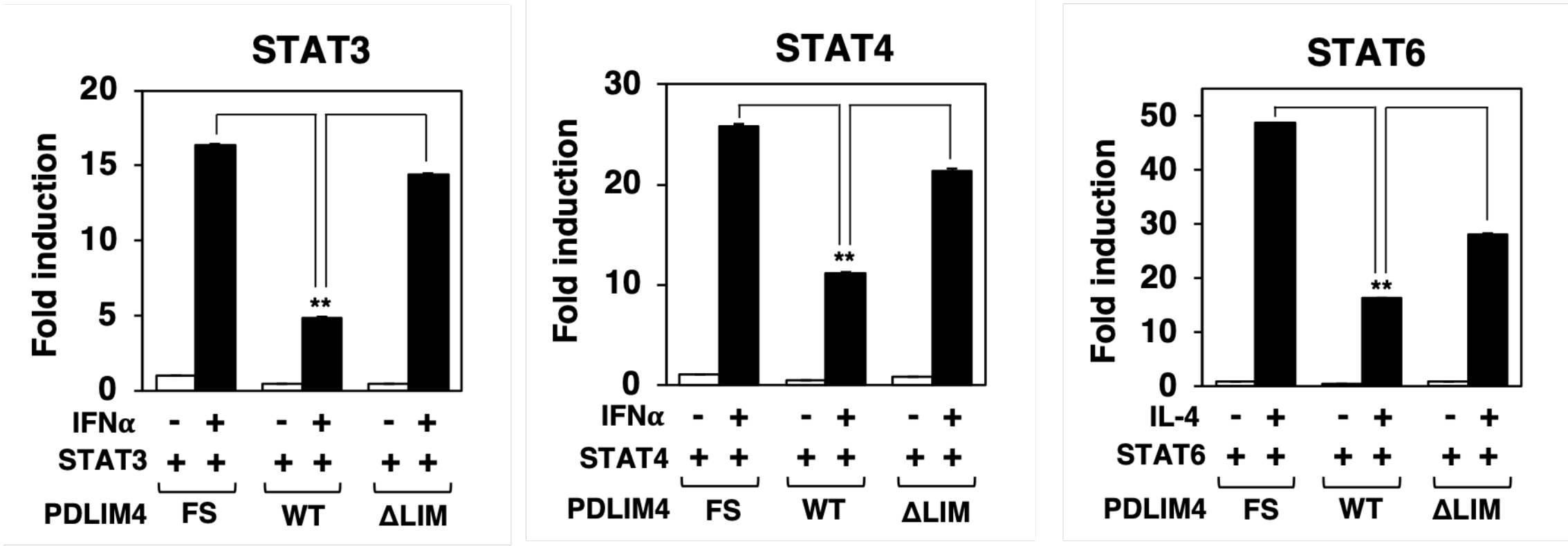

Supplementary Figure 1.

Luciferase activity in HEK293T cells transfected with the (2x) IRF-1 luciferase reporter plasmid without or with plasmids encoding STAT3 (left), STAT4 (center), or STAT6 (right) together with c-Myc-tagged wild-type or the indicated PDLIM4 mutants, stimulated with IFNα (1 ng/ml) or IL-4 (1 ng/ml) for 4 hours. Data are mean ± SD. **P < 0.01 and are representative of three independent experiments.

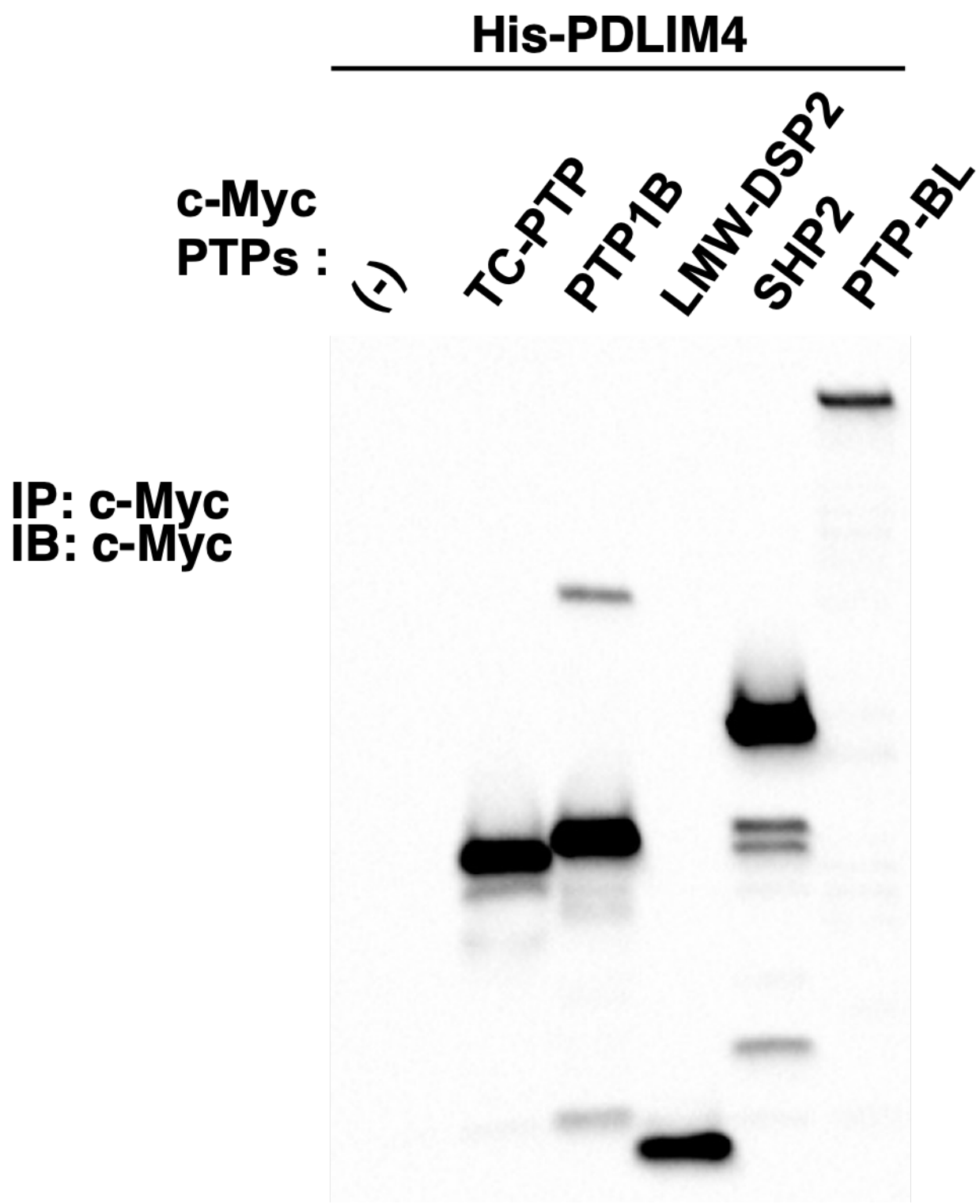

Supplementary Figure 2.

HEK293 T cells were transfected with plasmids encoding His-tagged PDLIM4 together without or with c-Myc-tagged TC-PTP, PTP1B, LMW-DSP2, SHP2, or PTP-BL. To confirm the expression of these PTPs, whole cell lysates were analyzed by Western blotting with anti-c-Myc antibody. Data are representative of four independent experiments.

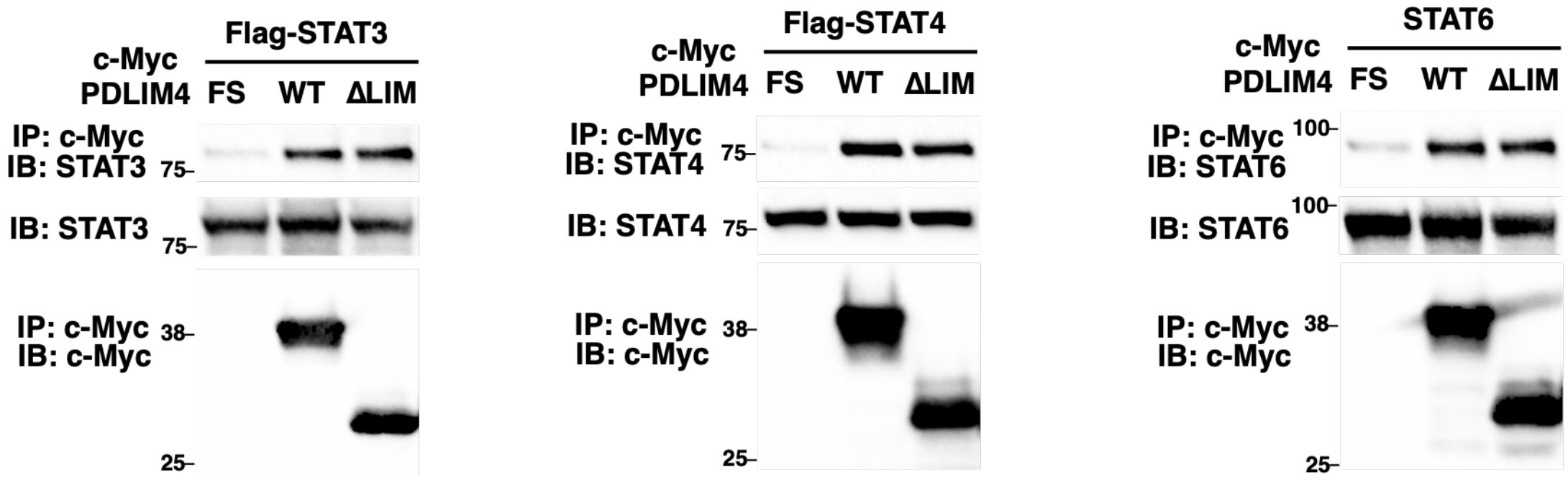

Supplementary Figure 3. HEK293T cells were transfected with plasmids encoding STAT3 (left), STAT4 (center), or STAT6 (right) together with c-Myc-tagged wild-type, frameshift or ΔLIM mutants of PDLIM4. Whole cell lysates were immunoprecipitated with a c-Myc antibody and analyzed by Western blotting with STAT3 (left), STAT4 (center), or STAT6 (right) antibodies. Data are representative of three independent experiments.

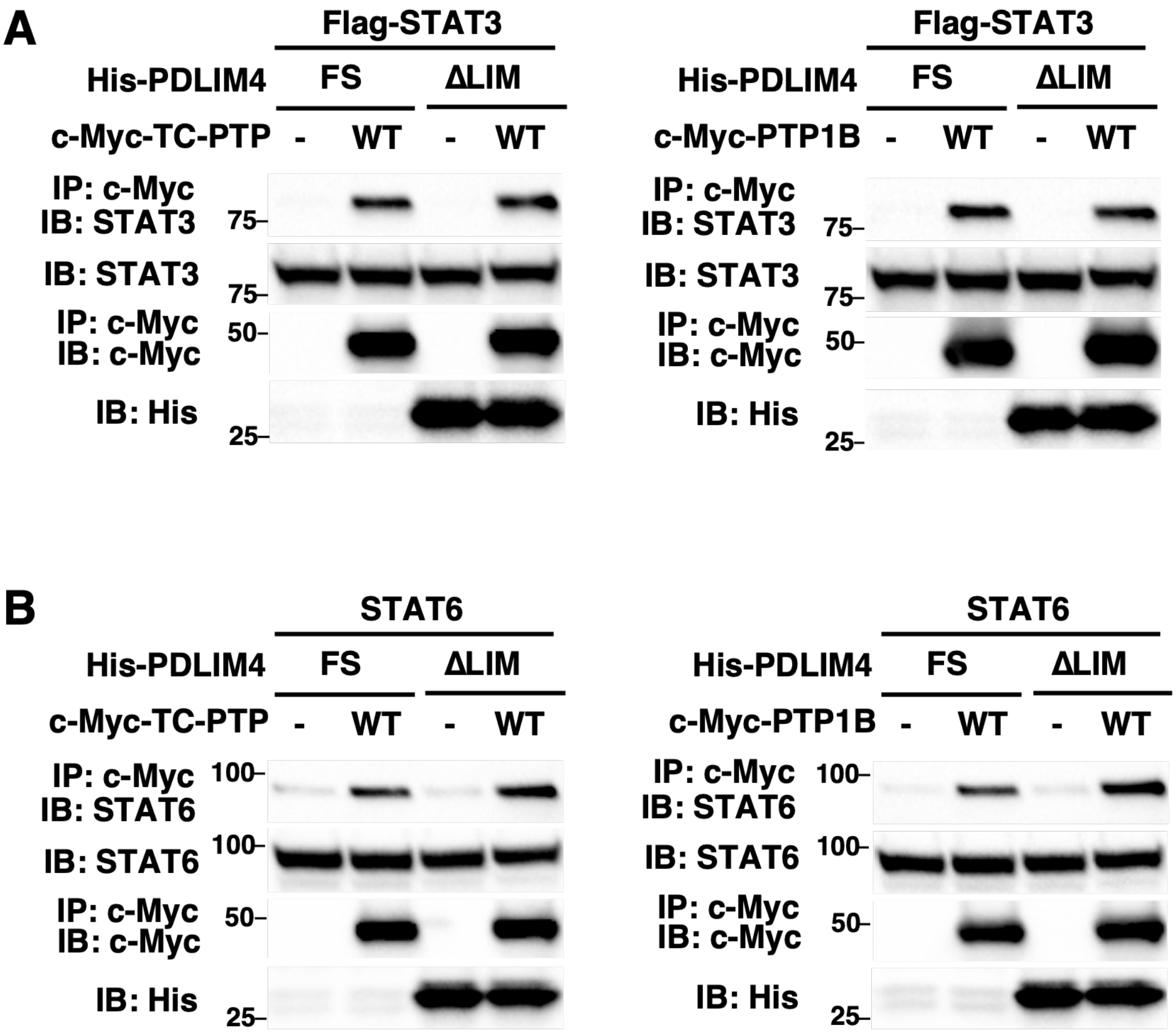

Supplementary Figure 4.

HEK293T cells were transfected with plasmids encoding STAT3 (A) or STAT6 (B) together without or with c-Myc-tagged TC-PTP (left) or PTP1B (right) in the absence or presence of the ΔLIM PDLIM4 mutant. Whole cell lysates were immunoprecipitated with a c-Myc antibody and analyzed by Western blotting with anti-STAT3 (A) or anti-STAT6 (B) antibodies. Data are representative of two independent experiments.

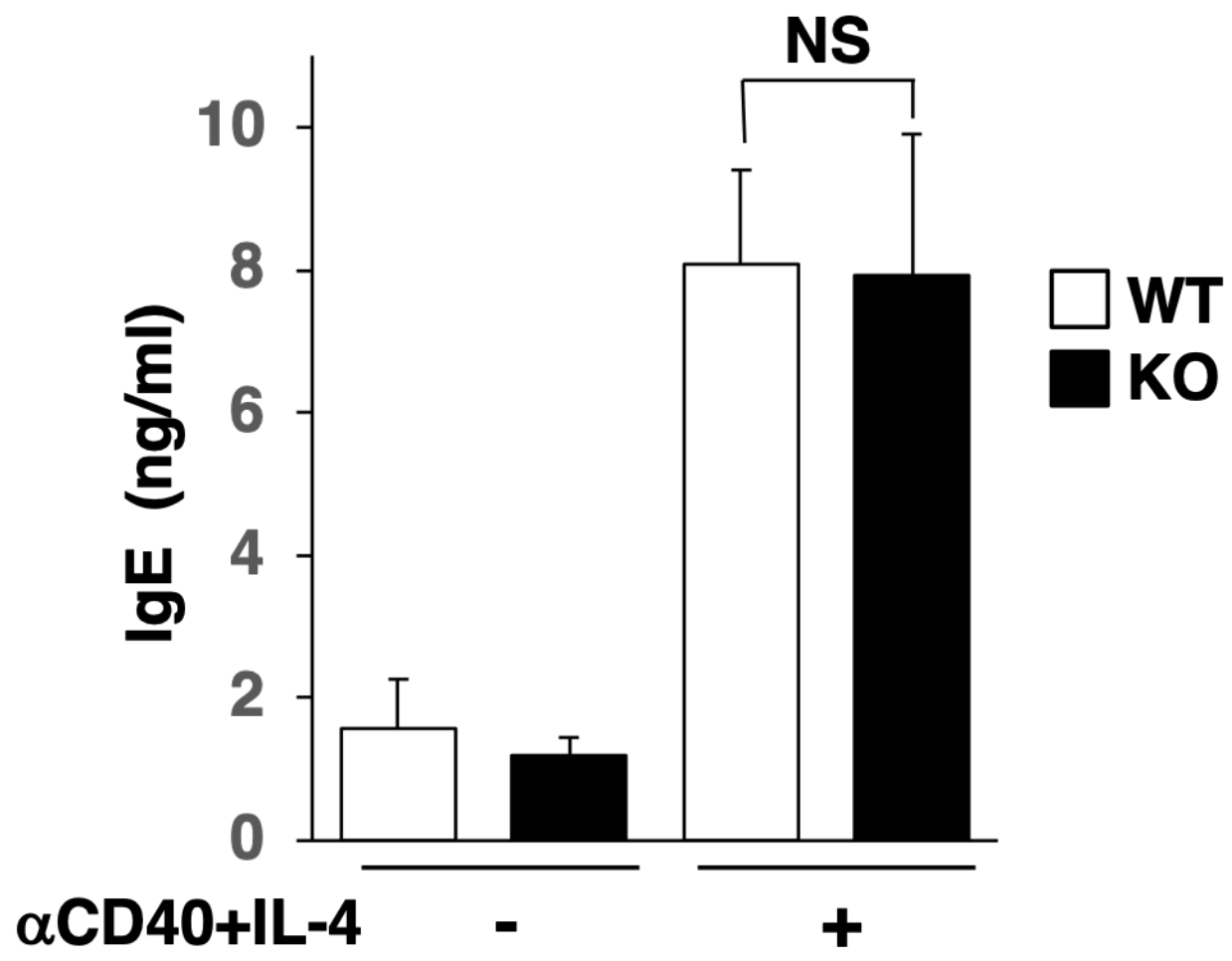

Supplementary Figure 5.

B cells purified from wild-type (n=3) and PDLIM4-deficient mice (n=3) were cultured with anti-CD40 plus IL-4 for 5 days. The production of IgE in the supernatants was measured by ELISA (means ± SD, NS; not significant).
